# Causes of death analysis and the prognostic model construction in neuroendocrine carcinoma of the cervix: A SEER‐based study

**DOI:** 10.1002/cam4.70066

**Published:** 2024-08-09

**Authors:** Haijuan Yu, Yongtian Lin, Jie Lin, Ning Xie, Linying Liu, Sufang Deng, Yang Sun

**Affiliations:** ^1^ Department of Gynecology Clinical Oncology School of Fujian Medical University, Fujian Cancer Hospital Fuzhou Fujian China; ^2^ Department of Epidemiology Clinical Oncology School of Fujian Medical University, Fujian Cancer Hospital Fuzhou Fujian China

**Keywords:** ADC, causes of death, NECC, risk factors, prognostic model, SCC

## Abstract

**Purpose:**

Neuroendocrine carcinoma of the cervix (NECC) is rare but results in poor prognosis. The causes of death (CODs) in NECC patients are rarely reported. Our study aimed to explore the distributions of death causes of NECC patients compared with squamous cell carcinoma (SCC) and adenocarcinoma (ADC) and to develop a validated survival prediction model.

**Methods:**

Patients diagnosed with NECC, SCC, or ADC were identified from the Surveillance, Epidemiology, and End Results Program database from 1975 to 2019. We analyzed the standardized mortality ratio (SMR) to determine each cause of death for each survival time category. The Kaplan–Meier method was used for survival analysis. Univariate and multivariate Cox regression analyses were used to establish a nomogram model.

**Results:**

A total of 358 NECC patients were included in this study, and 270 (75.4%) died during the follow‐up period. Patients with NECC had 5.55 times (95% CI, 4.53–6.79, *p* < 0.0001) higher risk of death compared with patients with SCC and 10.38 times (95% CI, 8.28–13.01, *p* < 0.0001) higher compared with ADC. Cervical cancer is the main cause of death in NECC. As the diagnosis time increased, the risk of death from all causes and cervix cancer gradually decreased. While after at least 10 years of follow‐up time, the highest and most dramatical SMR values were observed for metastasis (SMR, 138.81; 95% CI, 37.82–355.40; *p* < 0.05) and other cancers as the reason for death has an over 7‐fold higher SMR (SMR: 7.07; 95% CI: 2.60–15.40, *p* < 0.05) more than 5 years after the cancer diagnosis. Race, FIGO stage, and surgery were independent risk factors for the overall survival (OS) of NECC patients. For the predictive nomogram, the C‐index was 0.711 (95% CI: 0.697–0.725) and was corrected to 0.709 (95% CI: 0.680, 0.737) by bootstrap 1000 resampling validation.

**Conclusion:**

Compared with SCC and ADC, NECC patients have an elevated risk of mortality due to cervical cancer and metastasis. We successfully constructed a prognostic nomogram for patients with NECC. Based on refractoriness and high mortality of NECC, targeted treatment strategies and follow‐up plans should be further developed according to the risk of death and distribution characteristics of CODs.

## 1 INTRODUCTION

Neuroendocrine carcinoma of the cervix (NECC) is a rare variant of cervical cancer that accounts for 1%–1.5% of all cervical malignancies^1^, ^2^ but results in high fatality and psychological burden.^3^ The prognosis of NECC is poor,^4^ with a mean overall survival (OS) of 22–40 months and 5‐year cancer‐specific survival (CSS) rates of less than 30%–45%.^5^, ^6^ Even in the early stages, NECC patients exhibit high mortality and recurrence rates.^7^ The clinical manifestations of NECC are similar to those of other types of cervical cancer, such as irregular bleeding and pelvic pain. However, most patients have no neuroendocrine‐related symptoms such as Cushing syndrome,^8^ hypoglycemia, carcinoid syndrome, and visual impairment.^9^ There is no standardized therapy for this type of malignancy based on controlled trials,^10^ according to the Society of Gynecologic Oncology (SGO)and the Gynecologic Cancer Intergroup (GCIG) recommend a multimodality strategy including surgery, systemic chemotherapy (CHT), and radiotherapy (RT), which mainly based on the therapy of cervical cancer in general as well as from neuroendocrine tumors of the lung in particular.^1^ Despite the massive advancement in NECC treatment, its management still poses a real clinical challenge. Thus, effective models based on the large cohort that possesses the ability to predict the survival of patients with NECC and clinical trials designed for those with a high risk of recurrence and death are demanding.^11^

To our knowledge, few studies have investigated the distribution of causes of death (CODs) in NECC patients, leading to a dilemma in clinical care and follow‐up strategies for NECC patients. It is confusing that the management of NECC patients is more similar to extracervical neuroendocrine carcinoma (NEC, e.g. lung or gastro‐entero‐pancreatic NEC), or cervix cancer. Recent studies have shown that small cell neuroendocrine carcinoma of the cervix (SCNEC) is genetically more related to the phenotype of cervical cancer than that of the lung and bladder.^12^, ^13^ Schultheis AM et al. also found that small cell neuroendocrine carcinoma more closely resembled HPV‐driven cervical cancers, including squamous cell carcinoma (SCC) and cervical adenocarcinom (ADC).^14^ Based on these reasons, we explored the differences and similarities between the distribution of CODs in NECC patients and those of SCC and ADC to investigate the unique characteristics of NECC patients and provide guidance on follow‐up strategies.

In this study, we used data from the surveillance, epidemiology, and results cancer registration database (SEER) (1975–2019) to explore the changing trends of death causes of NECC patients compared with SCC and ADC and develop a validated survival prediction model to ameliorate the management plans and survival outcomes of patients with NECC.

## 2 MATERIALS AND METHODS

### 2.1 Patient

**FIGURE 1:**
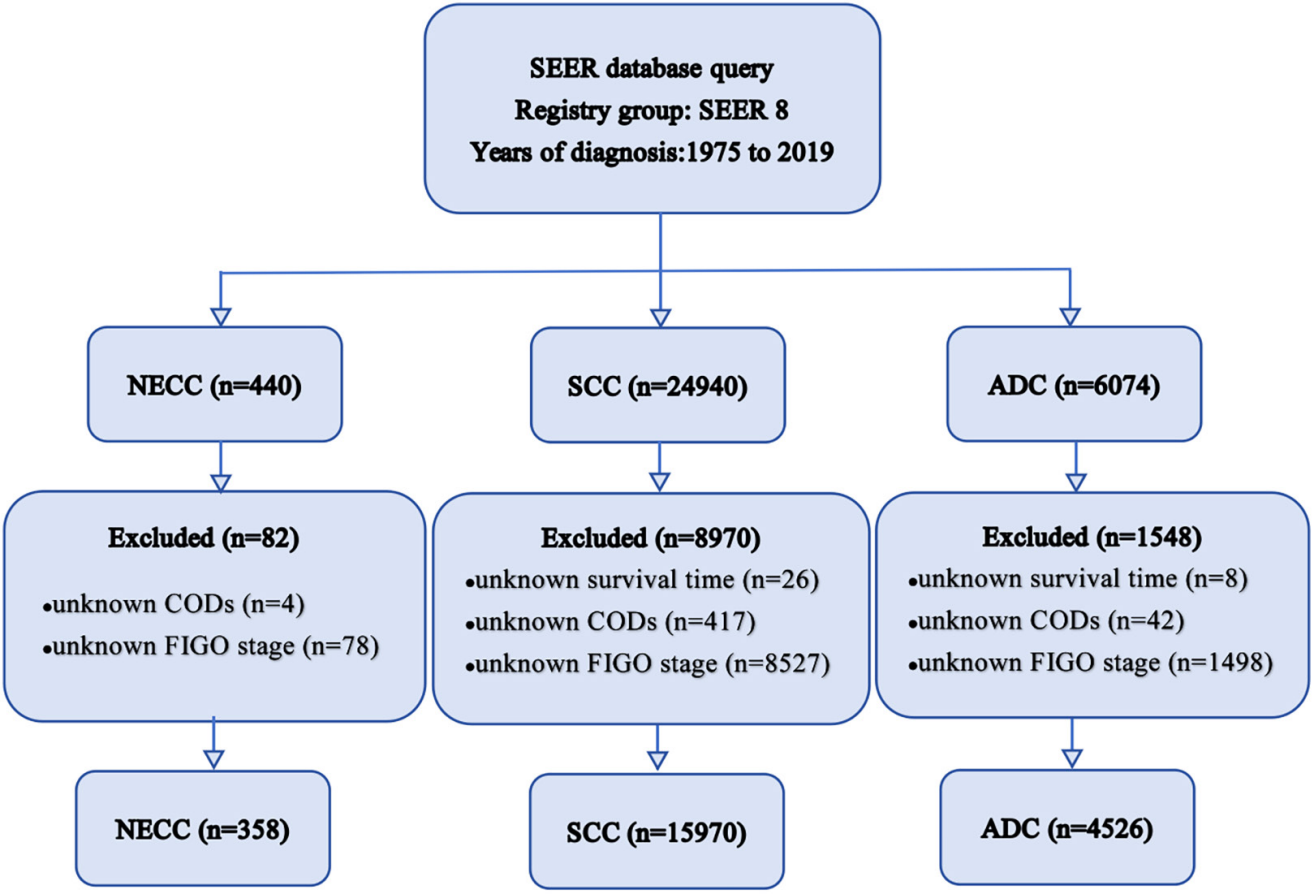
Patient extraction flowchart.

The SEER database is currently the largest publicly available cancer database, covering approximately 8.3% of the US population. The study data and related clinical information on NECC, SCC, and ADC were obtained from the SEER progra Incidence—SEER Research Plus Data, 8 Registries, Nov 2021 Sub (1975–2019) from SEER*Stat (version 8.4.1) (http://www.seer.cancer.gov). The following were selected: (1) C53.0‐Endocervix, C53.1‐Exocervix, C53.8‐Overlapping lesion of the cervix and C53.9‐Cervix uteri as the primary sites; (2) 1975–2019 for the year of diagnosis. We utilized the 2018 Federation Internationale de Gynecologie et d'Obstetrique (FIGO) stage system in this study. The exclusion criteria were as follows: (I) unknown survival time; (II) unknown CODs; (III) unknown FIGO stage. The screening scheme for the subjects is provided in Figure 1.

### 2.2 Data collection

The following data were collected, including year of diagnosis, age at diagnosis, race, pathological type, median household income, rural–urban Continuum Code, stage, tumor size, regional nodes involvement, distant metastasis, therapy method, vital status recode, survival months, CODs, SEER cause‐specific death classification and SEER other cause of death classification. SEER CODs are recorded based on the International Statistical Classification of Diseases and Related Health Problems, 10th Revision. In this study, CODs were categorized as cervix cancer death (death due to cervix cancer), metastasis death (SEER cause‐specific death classification is “dead [attributable to this cancer dx]”) but COD is cancer excluding cervix cancer, other cancer causes death (death due to cancer excluding cervix cancer, and SEER other cause of death classification is “dead [attributable to causes other than this cancer dx]”), and non‐cancer death (death due to any non‐cancer causes).

### 2.3 Ethics statement

Since the clinical data in this study was collected from a publicly available database, there were no local or state ethical issues, and informed consent was not required.

### 2.4 Statistical analysis

The chi‐squared test, Yates' correction, or Fisher exact test were used for comparing categorical variables, while the nonparametric rank sum test was utilized for comparing continuous variables. Continuous variables were transformed into categorical variables based on cut‐off points using the X‐tile software version 3.6.1, which is essential for generating the best cutoff point with the minimum *p*‐value. We calculated the survival rates between subgroups by Kaplan–Meier curves, and survival differences were examined by the log‐rank test. To describe the distribution of death causes of cervical cancer patients, we calculated the percentage within the latency period: <2 years, 2–5 years, and >5 years following a cervix cancer diagnosis. The standardized mortality ratio (SMR) and the 95% confidence interval (CI) of SMRs among each cause of death within each latency period were obtained using SEER Stat 8.4.3 software. We adopted SMRs to compare the variation in the risk of death for each cause among patients with cervix cancer and the US general population. The SMR is the observed‐to‐expected ratio, which reflects the strength of the correlation for each cause of death. This observation refers to the number of deaths from cervix cancer. Expected refers to the expected number of deaths in the general population. The general population refers to a population structure similar to cancer patients after adjusting age, race, sex, and year.^15^ For testing the significance of the observed SMRs, the simple continuity corrected chi‐square statistic was used to test whether the observed number of deaths is significantly different from the expected number. A SMR greater than 1 indicates a higher relative risk of each death causes in every pathological type of cervix cancer versus the general US population.^16^, ^17^, ^18^

Then, univariable and multivariable Cox proportional hazard regression analyses were used to estimate the risk factors related to the prognosis of NECC patients. Risk factors in multivariable analysis (*p* < 0.05) were selected to construct a prognostic nomogram. The concordance index (C‐index), and the receiver operating characteristic (ROC) curve calculated by bootstrapping was used to assess the performance. The calibration plot reflected the consistency between the predicted probability and the actual probability. DCA was calculated by the net benefit over a spectrum of probability thresholds (Details shown in Supplementary Materials—Data S1). All *p*‐values are two‐sided. To account for multiple comparisons, we also calculated Bonferroni *p* value thresholds for significance, *p* = 0.05 divided by the number of tests. All analyses were performed by STATA (version 26.0), R software (version 4.3.1), and GraphPad Prism (GraphPad 6.0.1).

## 3 RESULTS

### 3.1 Baseline characteristics

There were 358 patients with NECC, 15970 with SCC, and 4526 with ADC. Patients with NECC were more advanced stag (*p* < 0.001), and died at earlier age compared with patient (*p* < 0.001) with SCC and ADC. Patients who died of NECC were more likely to have a median household income greater than $75,000 compared with patients with SC (*p* < 0.001). The most common pathological type is SCNEC (66.5%). Nonetheless, tumor size ≥4 cm, regional lymph node metastasis, and distant metastasis were more frequent among patients with NECC, compared with ADC and SCC. NECC patients were prone to be treated with chemotherapy rather than surgery compared with ADC and SC (*p* < 0.001) (Table 1).

**TABLE 1:** Clinical and pathological characteristics of patients with NECC, SCC, and ADC.

Characteristic	NECC (*n* = 358)						SCC (*n* = 15,970)			ADC (*n* = 4526)		
	No. (%)	*p* value	Deaths No. (%)	*p* value	Age at deat (years) mean ± SD (*n* = 358)	*p* value	No. (%)	Deaths No. (%)	Age at deat (years) mean ± SD (*n* = 15,970)	No. (%)	Deaths No. (%)	Age at deat (years) mean ± SD (*n* = 4526)
Overall	358 (100.0)		270 (75.4)	**,**##**	52.5 ± 17.0	**,**##**	15,970 (100.0)	7354 (46.0)	63.1 ± 16.3	4526 (100.0)	1556 (34.4)	64.4 ± 15.7
Age at diagnosi (years)		*,**#**		**,**##**								
<40	127 (35.4)		84 (66.1)		34.0 ± 5.7	**,**##**	4695 (29.4)	993 (21.2)	39.8 ± 8.8	1397 (30.9)	182 (13.0)	40.4 ± 9.7
≥40	231 (64.5)		186 (37.2)		61.3 ± 13.2	**,**##**	11,275 (70.6)	6361 (56.4)	66.7 ± 14.1	3129 (69.1)	1374 (43.9)	67.6 ± 13.5
Race		*,**##**		*								
White	259 (72.3)		193(74.5)		51.3 ± 16.6	**	11,737 (73.5)	5369 (45.7)	63.3 ± 16.0	3617 (79.9)	1182 (32.7)	65.2 ± 15.6
Black	34 (9.5)		30 (88.2)		54.5 ± 16.0	**##**	2035 (12.7)	1121 (55.1)	60.5 ± 17.0	279 (6.2)	165 (59.1)	64.9 ± 15.5
Others	63 (17.6)		47 (74.6)		55.4 ± 18.2	**	2083 (13.0)	855 (41.0)	65.2 ± 17.2	591 (13.1)	208 (35.2)	59.6 ± 15.8
Unknown	2 (0.6)		0 (0.0)		‐	‐	115 (0.7)	9 (7.8)	61.2 ± 17.0	39 (0.9)	1 (2.6)	57.3
Median household income		*		**								
<$75,000	160 (44.7)		117 (73.1)		49.9 ± 17.4	**,**##**	7970 (49.9)	3484 (43.7)	62.0 ± 16.2	2147 (47.4)	720 (33.5)	64.6 ± 15.9
≥$75,000	165 (46.1)		123 (74.5)		54.0 ± 15.3	**,**##**	6254 (39.2)	2579 (41.2)	63.8 ± 16.5	2045 (45.2)	606 (29.6)	63.5 ± 15.9
Unknown	33 (9.2)		30 (90.9)		56.8 ± 20.6	**,**#**	1746 (10.9)	1291 (73.9)	64.5 ± 16.1	334 (7.4)	230 (68.9)	66.0 ± 14.4
Rural–Urban Continuum Code				*								
Counties in metropolitan areas	276 (77.1)		200 (72.5)		52.1 ± 16.1	**,**##**	11,796 (73.9)	4905 (41.6)	62.6 ± 16.4	3539 (78.2)	1064 (30.1)	65.5 ± 15.1
Nonmetropolitan counties	45 (12.6)		37 (82.2)		51.9 ± 18.8	**,**##**	2110 (13.2)	1005 (47.6)	63.3 ± 16.3	564 (12.5)	221 (39.2)	66.0 ± 15.8
Unknown	37 (10.3)		33 (89.2)		55.9 ± 20.1	*,**#**	2064 (12.9)	1444 (70.0)	64.8 ± 16.1	423 (9.3)	271 (64.1)	65.5 ± 15.1
Pathological type		‐		‐								
SCNEC	238 (66.5)		181 (76.1)		53.3 ± 17.4	‐	‐	‐	‐	‐	‐	‐
Non‐SCNEC	25 (7.0)		18 (72.0)		46.8 ± 11.0	‐	‐	‐	‐	‐	‐	‐
Unknown	95 (26.5)		71 (74.7)		52.0 ± 17.2	‐	‐	‐	‐	‐	‐	‐
FIGO stage		**,**##**		**,**##**								
I	105 (29.3)		58 (55.2)		50.8 ± 16.9	**,**##**	8198 (51.3)	2217 (27.0)	67.0 ± 16.7	3050 (67.4)	584 (19.1)	67.7 ± 16.3
II	27 (7.5)		23 (85.2)		61.9 ± 21.8		2307 (14.4)	1316 (57.0)	64.6 ± 16.5	391 (8.6)	206 (52.7)	63.9 ± 15.9
III	92 (25.7)		70 (76.1)		49.2 ± 15.3	**,**##**	3128 (19.6)	1777 (56.8)	60.9 ± 16.1	509 (11.2)	290 (57.0)	61.3 ± 16.3
IV	134 (37.4)		119 (88.8)		53.5 ± 16.4	**,**##**	2337 (14.6)	2044 (87.5)	59.9 ± 14.9	576 (12.7)	476 (82.6)	62.4 ± 13.8
Tumor size (cm)		**,**##**		**#**								
<4	75 (20.9)		45 (60.0)		44.9 ± 13.2	**,**##**	6507 (40.7)	1522 (23.4)	65.2 ± 16.6	2131 (47.1)	367 (17.2)	64.4 ± 15.8
≥4	111 (31.0)		86 (77.5)		49.7 ± 16.5	**,**##**	3277 (20.5)	1991 (60.8)	58.8 ± 15.6	640 (14.1)	381 (59.5)	60.4 ± 16.3
Unknown	172 (48.0)		139 (80.8)		56.8 ± 17.2	**,**##**	6186 (38.7)	3841 (62.1)	64.4 ± 16.2	1755 (38.8)	808 (46.0)	66.3 ± 15.1
Regional lymph node involvement		**,**##**		**,**##**								
Yes	122 (34.1)		95 (77.9)		47.8 ± 15.0	**,**##**	2560 (16)	1368 (53.4)	56.2 ± 14.8	560 (12.4)	330 (58.9)	58.1 ± 15.0
NO	138 (38.5)		89 (64.5)		54.2 ± 18.5	**,**##**	9911 (62.1)	3489 (35.2)	65.2 ± 16.5	3394 (75.0)	794 (23.4)	65.9 ± 15.9
Unknown	98 (27.4)		86 (87.8)		56.1 ± 16.3	**,**##**	3499 (21.9)	2497 (71.4)	64.0 ± 15.9	572 (12.6)	432 (75.5)	66.3 ± 14.8
Distant metastasis		**,**##**		**,**##**								
Yes	132 (36.9)		118 (43.7)		53.8 ± 16.2	**,**##**	2213 (13.9)	1956 (26.6)	59.7 ± 14.9	560 (12.4)	464 (29.8)	62.2 ± 13.8
No	226 (63.1)		152 (56.3)		51.6 ± 17.6	**,**##**	13,757 (86.1)	5398 (73.4)	64.3 ± 16.6	3966 (87.6)	1092 (70.2)	65.3 ± 16.4
Metastatic sites												
Bone	12 (2.3)		11 (91.7)		53.4 ± 18.6		98 (0.6)	73 (74.5)	55.4 ± 12.3	32 (0.7)	24 (75.0)	59.9 ± 12.6
Brain	2 (0.4)		1 (50.0)		53.4		12 (0.7)	12 (100)	50.0 ± 12.1	5 (0.1)	4 (80.0)	53.3 ± 16.2
Liver	18 (3.5)		16 (88.9)		49.3 ± 15.3	**#**	75 (0.5)	68 (90.7)	55.0 ± 12.9	28 (0.6)	25 (89.3)	61.3 ± 15.5
Lung	21 (4.1)		17 (81.0)		50.0 ± 16.4	**#**	160 (1.0)	132 (82.5)	57.5 ± 13.6	46 (1.0)	35 (76.1)	62.5 ± 14.2
Distant lymph node	12 (2.3)		11 (91.7)		54.3 ± 15.4		132 (0.8)	79 (59.8)	57.5 ± 14.4	34 (0.8)	26 (76.5)	59.2 ± 13.9
Others	8 (1.6)		7 (87.5)		54.4 ± 15.4		55 (0.3)	39 (70.9)	61.6 ± 17.0	38 (0.8)	20 (52.6)	61.1 ± 14.9
Surgery		**,**##**		**##**								
Yes	170 (47.5)		109 (64.1)		46.4 ± 14.6	**,**##**	9351 (58.6)	2788 (29.8)	62.8 ± 16.8	3437 (75.9)	789 (23.0)	63.4 ± 16.2
No/Unknown	188 (52.5)		161 (85.6)		56.8 ± 17.4	**,**##**	6619 (41.5)	4566 (69.0)	63.3 ± 16.0	1089 (23.4)	767 (70.4)	65.4 ± 15.1
Radiotherapy		**##**		**								
Yes	207 (57.8)		160 (77.3)		51.6 ± 17.7	**,**##**	8613 (53.9)	5251 (61.0)	62.2 ± 16.2	1765 (39.0)	956 (54.2)	63.7 ± 16.0
No/Unknown	151 (42.2)		110 (72.8)		53.9 ± 15.9	**,**##**	7357 (46.1)	2103 (28.6)	65.3 ± 16.4	2761 (61.0)	600 (21.7)	65.5 ± 15.3
Chemotherapy		**,**##**		**,**##**								
Yes	261 (72.9)		193 (73.9)		49.1 ± 14.9	**,**##**	5247 (32.9)	2732 (52.1)	57.0 ± 14.5	1168 (25.8)	575 (49.2)	58.5 ± 13.9
No/Unknown	97 (27.1)		77 (79.4)		61.0 ± 18.9	*,**##**	10,723 (67.1)	4622 (43.1)	66.7 ± 16.3	3358 (74.2)	981 (29.2)	67.8 ± 15.7

*Note*: SCC vs. NECC. Bold *p*‐values: Indicates *p*‐values that remain significant at the Bonferroni threshold.

Abbreviation: ‐, not calculated.

* *p* < 0.05.

** *p* < 0.01, ADC vs. NECC.

^#^ *p* < 0.05.

^##^ *p* < 0.01.

### 3.2 CODs characteristics

**FIGURE 2:**
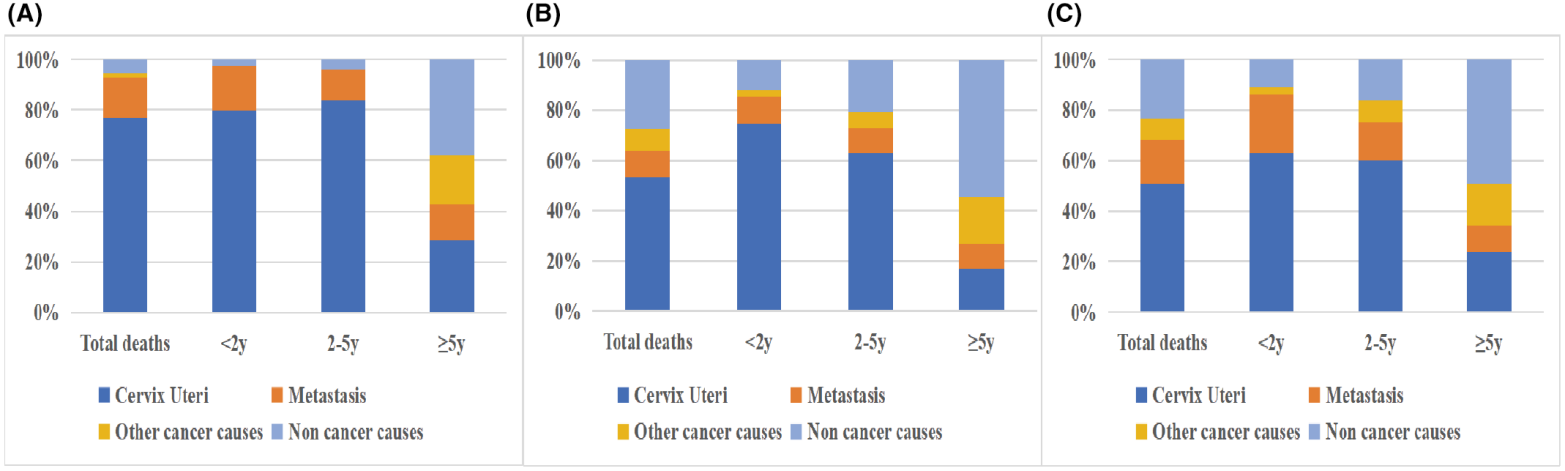
CODs of different histological types of cervix cancer within each latency period: (A) NECC (B) SCC and (C) ADC.

We explored the CODs of cervical cancer patients with three histological types in different time stratifications. 76.7% of the 270 NECC patients who died were caused by NECC itself, followed by metastasis (16.3%), non‐cancer causes (5.6%), and other cancer cause (1.5%). Compared with SCC and ADC patients, the majority of NECC patients (73.7%) died of all causes within 2 years after diagnosed (*p* < 0.001). Death from cervical cancer in NECC patients also occurs primarily within 2 years of diagnosis (*p* < 0.001). Patients with NECC who died from cervical cancer metastasis had a significant difference in the distribution of time to death compared with SCC (*p* < 0.001), but not ADC (Table 2, Figure 2).

**TABLE 2:** Death causes of three types of cervical cancers in different time stratifications.

Cause of death	NECC deaths by time after diagnosis (*n* = 358) No. (%)					SCC deaths by time after diagnosis (*n* = 15,970) No. (%)				ADC deaths by time after diagnosis (*n* = 4526) No. (%)			
	Total deaths	<2 years	2–5 years	≥5 years	*p* value	Total deaths	<2 years	2–5 years	≥5 years	Total deaths	<2 years	2–5 years	≥5 years
All					**,**##**								
	270 (100)	199 (73.7)	50 (18.5)	21 (7.8)		7354 (100)	3462 (47.1)	1502 (20.4)	2390 (32.5)	1556 (100)	775 (49.8)	318 (20.4)	463 (29.8)
Cervix Uteri					**,**##**								
	207 (76.7)	159 (76.8)	42 (20.3)	6 (2.9)		3936 (53.5)	2591 (65.8)	943 (24.0)	402 (10.2)	788 (50.6)	488 (61.9)	190 (24.1)	110 (14.0)
Metastasis					**								
	44 (16.3)	35 (79.5)	6 (13.6)	3 (6.8)		753 (10.2)	365 (48.5)	150 (19.9)	238 (31.6)	277 (17.8)	179 (64.6)	49 (17.7)	49 (17.7)
Other cancer causes													
	4 (1.5)	0 (0.0)	0 (0.0)	4 (100.0)		641 (8.7)	93 (14.5)	100 (15.6)	448 (69.9)	127 (8.2)	23 (18.1)	28 (22.0)	76 (59.8)
Non cancer causes													
	15 (5.6)	5 (31.3)	2 (12.5)	8 (53.3)		2024 (27.5)	413 (20.4)	309 (15.3)	1302 (64.3)	364 (23.4)	85 (23.4)	51 (14.0)	228 (62.6)

*Note*: SCC vs. NECC. Bold *p*‐values: Indicates *p*‐values that remain significant at the Bonferroni threshold.

* *p* < 0.05.

** *p* < 0.01,ADC vs. NECC.

^#^ *p* < 0.05.

^##^ *p* < 0.01.

### 3.3 Survival curves

**FIGURE 3:**
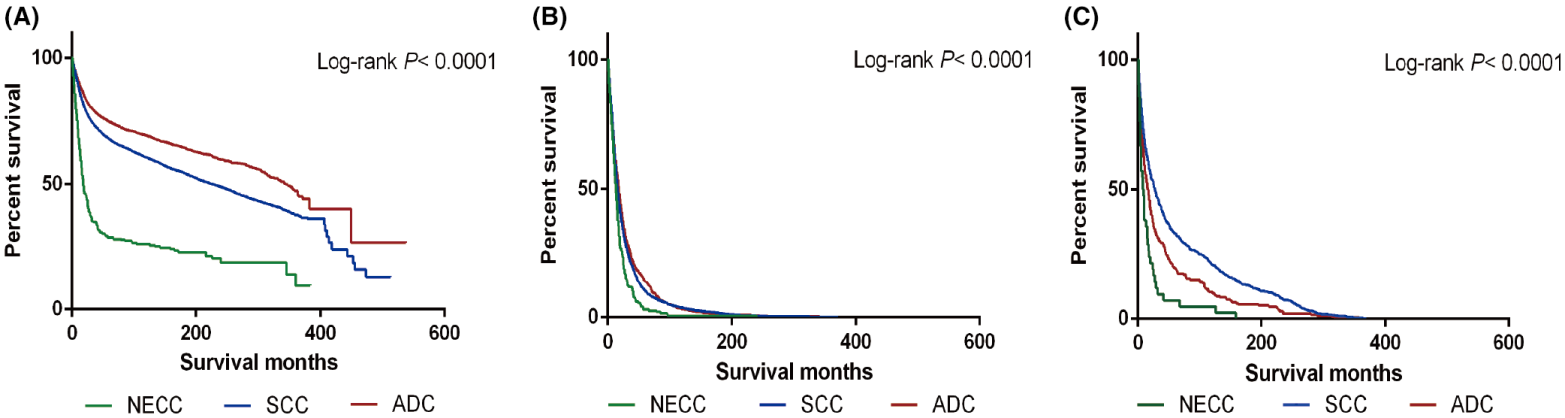
Survival curves by histological type: (A) overall; (B) cervix uteri; (C) metastasis.

Figure 3 shows survival curves according to histological type and CODs. Mortality of patients with NECC was significantly different from that of patients with SCC (HR 5.55, 95% CI, 4.53–6.79, *p* < 0.0001) and ADC (HR 10.38, 95% CI, 8.28–13.01, *p* < 0.0001) (Figure 3A). NECC patients had 1.46 times (95% CI, 1.24–1.73, *p* < 0.0001) higher risk of cervix cancer death compared with patients with SCC and 1.60 times (95% CI, 1.328–1.916, *p* < 0.0001) higher risk with patients with ADC (Figure 3B). The risk of dying from metastasis in NECC patients was 1.907 times (95% CI, 1.269–2.867, *p* < 0.01) higher than that in SCC patients and 3.970 times (95% CI, 2.473–6.373, *p* < 0.0001) higher than in ADC patients (Figure 3C).

### 3.4 The changes in SMR

**FIGURE 4:**
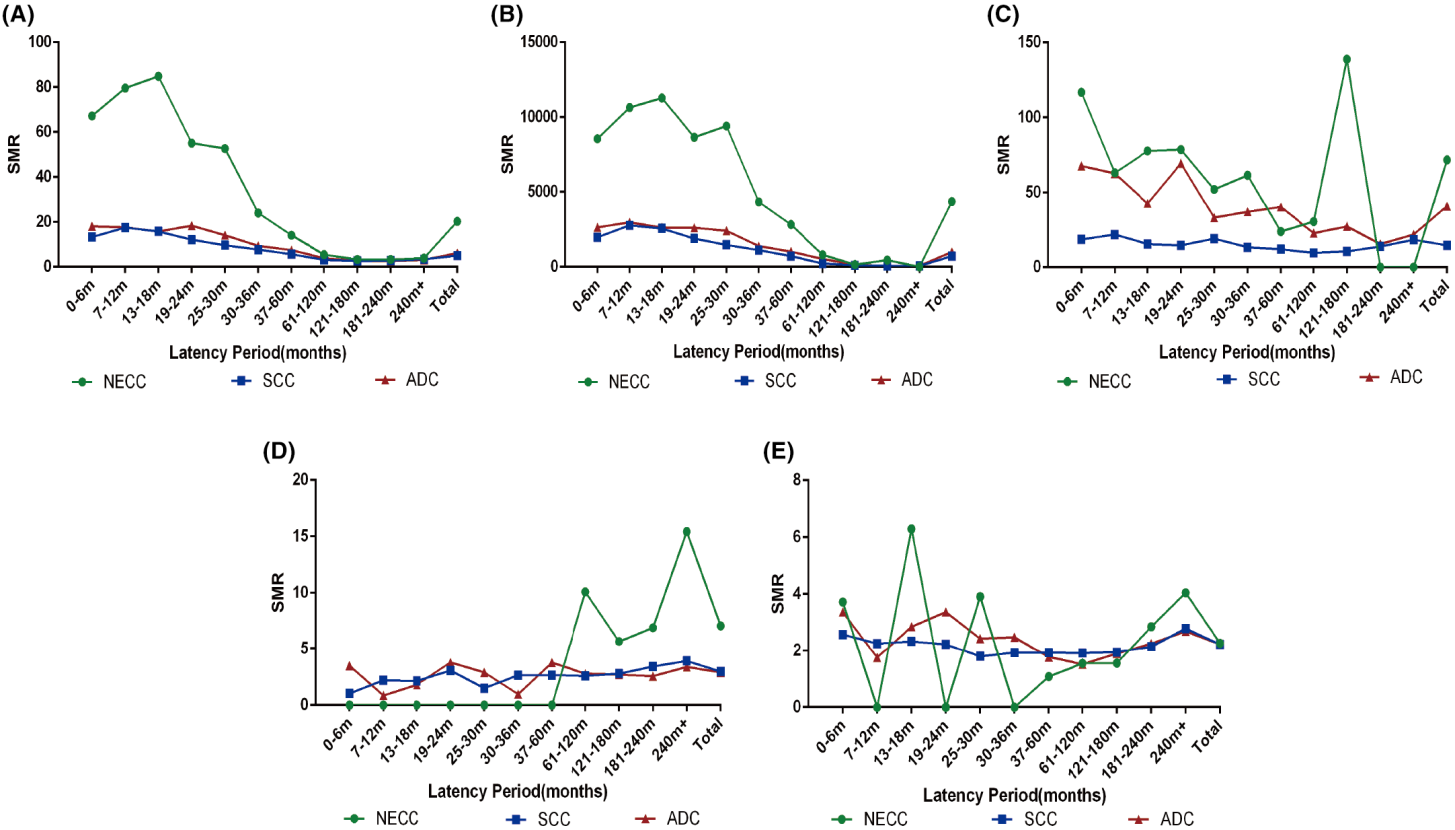
The changes in SMR of three histological types of cervical cancers with different causes of death and incubation periods. (A) overall; (B) cervix uteri;( C) metastasis; (D) other cancers causes; and (E) non cancer causes.

The risk of death from all causes (SMR: 20.31; 95% CI: 18.10–22.71, *p* < 0.05) (Figure 4A) and cervix cancer (SMR: 4370.02; 95% CI: 4370.02–4370.02, *p* < 0.05) (Figure 4B) was statistically significantly higher within 10 years of diagnosis. After at least 10 years of follow‐up time, the highest and most dramatical SMR values were observed for metastasis (SMR, 138.81; 95% CI, 37.82–355.40; *p* < 0.05)(Figure 4C). It is worth mentioning that other cancers as reasons for death have an over 7‐fold higher SMR (SMR: 7.07; 95% CI: 2.60–15.40, *p* < 0.05) more than 5 years after the cancer diagnosis compared to the general US population, which was also higher than SC (SMR: 2.98; 95% CI: 2.80–3.15, *p* < 0.05) and ADC patient (SMR: 2.91; 95% CI: 2.44–3.44, *p* < 0.05) (Figure 4D). Compared with the US general population, the risk of death from noncancer causes of NECC patients was higher (SMR: 2.27; 95% CI: 1.44–3.41), but similar to SC (SMR: 2.22; 95% CI: 2.15–2.29, *p* < 0.05) and AD (SMR: 2.23; 95% CI: 2.05–0.02, *p* < 0.05) (Figure 4E).

### 3.5 Cox proportional hazards regression model

To further investigate the variables that had varying impacts on the overall survival (OS) of patients with NECC. We conducted an analysis using a Cox proportional hazards regression model (Tables 2 and 3). There is collinearity between the FIGO stage and distant metastasi (Tables S1 and S2). Therefore, distant metastasis was not included in the analysis. The results revealed that race (Black individuals) [HR (95% CI): 1.520 (1.030–2.243), *p* = 0.035], FIGO stage III [HR (95% CI): 1.945 (1.201–3.150), *p* = 0.007], FIGO stage IV [HR (95% CI): 3.025 (2.002–4.572), *p* < 0.001], and surgery [HR (95% CI): 1.677 (1 0.245–0.2 0.260), *p* = 0 0.001] were identified as independent risk factors for the OS of NECC patients. Similar conclusions can be drawn if the FIGO stage does not include the analysis (Tables S3 and S4; Figure S1).

**TABLE 3:** Univariate and multivariate analysis of overall survival of NECC.

	Univariate analysis		Multivariate analysis	
	HR (95% CI)	*p* value	HR (95% CI)	*p* value
Age (years)				
<40	Reference		Reference	
≥40	1.652 (1.277–2.138)	< 0.001	1.301 (0.984–1.719)	0.064
Race				
White	Reference			
Black	1.716 (1.165–2.527)	0.006	1.520 (1.030–2.243)	0.035
Others	0.973 (0.707–1.339)	0.866	1.006 (0.723–1.398)	0.973
Unknown	0.000 (0.000–Inf)	0.939	0.000 (0.000–Inf)	0.947
Pathological type				
SCNEC	Reference			
Non‐SCNEC	0.980 (0.604–1.592)	0.936		
Unknown	1.009 (0.766–1.329)	0.948		
Median household income				
<$75,000	Reference			
≥$75,000	1.114 (0.864–1.435)	0.406		
Unknown	1.317 (0.871–1.990)	0.192		
Rural–Urban continuum code		0.353		
Counties in metropolitan areas	Reference			
Nonmetropolitan counties	1.135 (0.799–1.612)	0.479		
Unknown	1.232 (0.843–1.800)	0.280		
FIGO stage				
I	Reference		Reference	
II	1.806 (1.113–2.930)	0.017	1.149 (0.674–1.960)	0.609
III	2.351 (1.651–3.349)	< 0.001	1.945 (1.201–3.150)	0.007
IV	4.427 (3.200–6.125)	< 0.001	3.025 (2.002–4.572)	< 0.001
Tumor size				
≤4 cm	Reference		Reference	
>4 cm	1.795 (1.250–2.577)	0.006	0.983 (0.650–1.485)	0.934
Unknown	2.328 (1.660–3.263)	< 0.001	1.239 (0.835–1.840)	0.287
Regional lymph node involvement				
Yes	Reference		Reference	
No	0.518 (0.387–0.693)	< 0.001	1.013 (0.703–1.460)	0.944
Unknown	1.127 (0.840–1.512)	0.426	0.954 (0.677–1.345)	0.789
Distant metastasis				
Yes	Reference		‐	
No	0.335 (0.261–0.439)	< 0.001	‐	‐
Surgery				
Yes	Reference		Reference	
No/Unknown	2.256 (1.808–2.815)	< 0.001	1.677 (1.245–2.260)	0.001
Radiotherapy				
Yes	Reference			
No/Unknown	1.025 (0.803–1.308)	0.842		
Chemotherapy				
Yes	Reference			
No/Unknown	1.181 (0.903–1.545)	0.224		

Abbreviation: Inf, infinity; −, not calculated.

### 3.6 Development and validation of a nomogram

**FIGURE 5:**
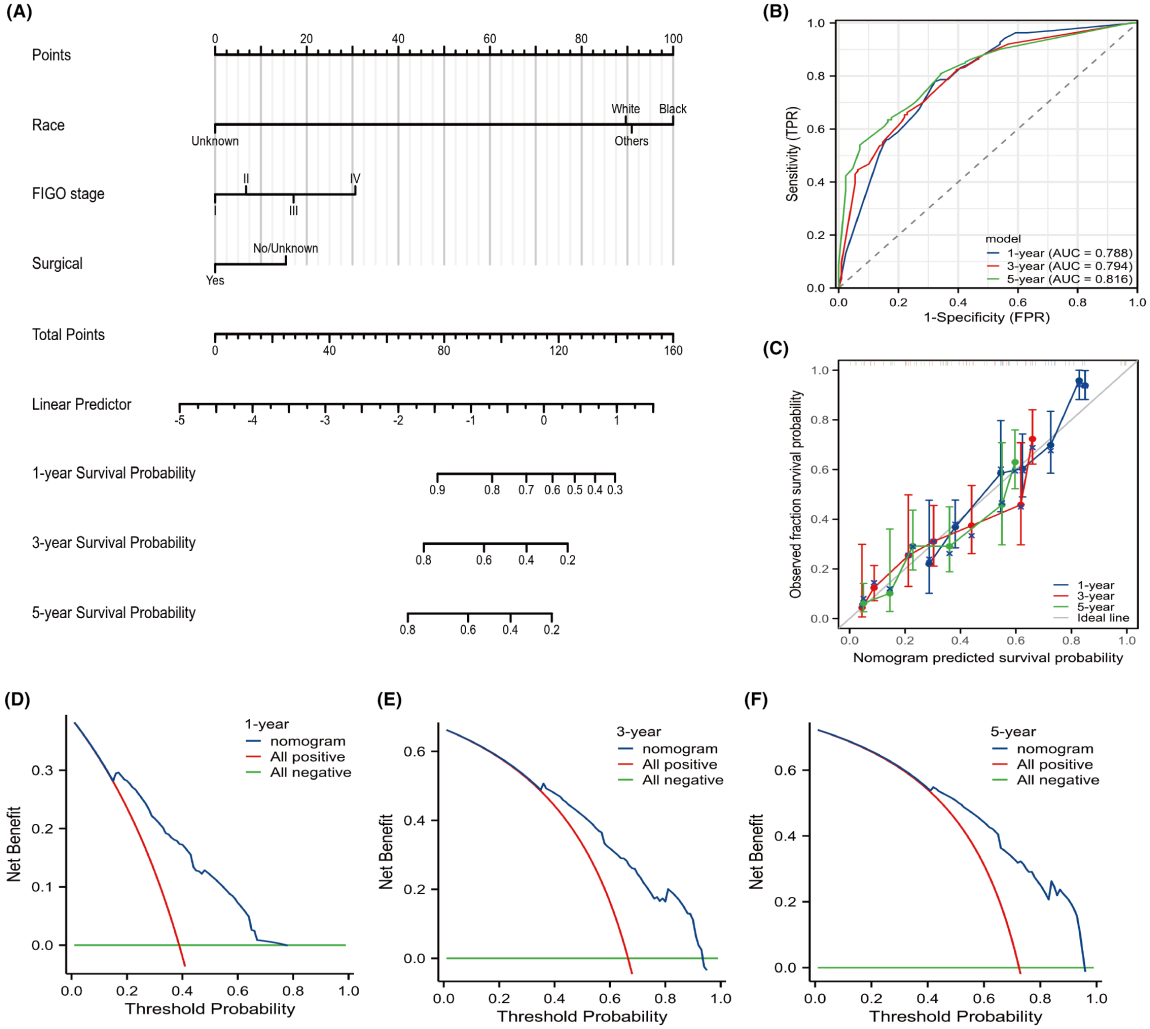
The construction and validation of nomograms. (A) Nomogram model predicting the 1‐, 3‐ and 5‐year OS in NECC patients. The nomogram is used by summing all points identified on the scale for each variable. The total points projected on the bottom scales indicate the probabilities of 1‐, 3‐ and 5‐year survival. (B) ROC curves for predicting 1‐, 3‐ and 5‐year OS. (C) The calibration curves for predicting 1‐, 3‐ and 5‐year OS in NECC patients. (D‐F) Decision curve analysis (DCA) for the nomograms in the prediction of 1‐, 3‐ and 5‐year OS in NECC patients.

Race, FIGO stage, and surgery were selected to formulate a nomogram to predict the OS of NECC patients (Figure 5A). The AUC values for 1‐, 3‐, and 5‐year were 0.788, 0.794, and 0.816 (Figure 5B). The C‐index for the prediction model was 0.711 (95% CI: 0.697–0.725) and was corrected to 0.709 (95% CI: 0.680, 0.737) by bootstrap 1000 resampling validation, which suggested a good discriminatory ability. The calibration curves of the nomograms showed good agreement between the predictive risk and the observed probability of 1‐, 3‐ and 5‐year OS (Figure 5C). Additionally, the DCA exhibited significantly better net benefits in nomograms among 1‐, 3‐ and 5‐year OS probabilities, indicating a greater potential for clinical decision making (Figure 5D–F).

## 4 DISCUSSION

NECC is an uncommon but aggressive uterine malignancy.^19^, ^20^, ^21^ However, to our knowledge, few studies currently focus on NECC death causes and recommendations for follow‐up strategies.^22^ Our study analyzed the characteristics and the changing trends of death causes for NECC patients and made a comparison with SCC and ADC patients. We found that the leading CODs for NECC patients were cervical cancer itself and metastasis. The risk was higher and longer‐lasting than that of SCC and ADC. We also found race, FIGO stage, and surgery are the independent prognostic factors for NECC patients. The nomogram we built is reliable and stable for prognostic prediction, which provides essential guidance and help for the health maintenance of NECC patients.

The biology of NECC differs from that of SCC or ADC in that it exhibits a very aggressive biological behavior with a strong propensity for lymphatic and hematogenous spread,^23^ so the clinical manifestation is usually local or distant metastasis.^24^, ^25^ The clinical management for NECC patients is similar with small cell lung cancer (SCLC). Even when combined with surgery, chemoradiation, and systemic chemotherapy, the prognosis remains dismal.^26^ As our study found, patients of NECC are more prone to local or distant metastasis and have poorer survival rates than other pathological types of cervical cancer. The higher incidence of metastasis in NECC patients still poses formidable challenges.^8^, ^27^ Monitoring for metastasis in other organs should be taken seriously to ensure proper management of patients with NECCs. The key player role of [18F] fluoro‐2‐deoxy‐D‐glucose (18F‐FDG) PET/CT during the follow‐up period should be emphasized in NECC patients. A NeCTuR study showed a CT scan was inferior to a PET/CT scan in assessing metastatic disease in high‐grade NECC patients.^28^ A large body of literature conveys that PET/CT is more valuable in the primary staging and detection of metastatic lesions for various malignancies, such as breast cancer, bladder cancer, and soft‐tissue or bone sarcoma.^29^, ^30^, ^31^ Using 18F‐FDG PET/CT during the follow‐up may contribute to NECC patient management.

Is it really scientific to follow the same approach for NECC, which has very different biological behavior and prognostic outcomes than SCC and ADC? In this study, we described the time trend of specific CODs in NECC patients. We found that the majority of NECC patients died of cervical cancer and metastasis within 2 years after diagnosis. However, the main CODs for NECC patients were not cervical cancer itself after a five‐year diagnosis. A similar result was also observed in pulmonary large cell neuroendocrine carcinoma and SCLC.^32^, ^33^ More importantly, when compared to SCC and ADC patients, the risk ratio of dying from cervical cancer and other cancers remained higher for NECC patients after a 5–10 year diagnosis. What's more, the highest and most dramatical SMR values were observed for metastasis after at least 10 years of follow‐up time. All of the above suggests the importance of general examination in follow‐up treatment and unique follow‐up principles for NECC patients. Although no definitive agreement exists on the best post‐treatment surveillance of cervical cancer, a reasonable follow‐up schedule involves follow‐up visits every 3–6 months in the first 2 years and every 6–12 months in years 3–5. Patients should return to annual population‐based general physical and pelvic examinations after 5 years of recurrence‐free follow‐up.^34^, ^35^ However, according to our findings, NECC patients should be followed up more frequently in the first 2 years, and the follow‐up time should be extended beyond 5 years to 10 years.

Our study revealed that the mortality risk of NECC is significantly higher than that of SCC and ADC, so it is necessary to further investigate the variables that had varying impacts on OS of patients with NECC. In our nomogram, the combination of race, FIGO stage, and surgery precisely predicts overall survival, especially 5‐year OS of NECC patients. It is worth noting that surgery may improve the outcomes of patients with NECC. A previous study showed patients with locally advanced disease or stage IB3‐IIA2 cancer might benefit from surgery.^36^ Zhang et al. also confirmed radical surgery followed by chemotherapy may be a favorable alternative intervention for selected patients with advanced stage cancer.^37^ Caruso G et al found that surgery after NACT for locally advanced NECC may yield similar outcomes compared to CRT.^38^ Although guidelines recommend non‐surgical methods as first‐line treatment, the application of surgery in NECC patients deserves further exploration.

There are some limitations in this study. First, the study was retrospective and selection bias existed. Secondly, due to the inherent weaknesses of the SEER database, we do not have included detailed information such as disease recurrence, postoperative complications, chemotherapy regimens, and chronology of surgery and RT, which were proved to have influences on the survival outcomes and may complicate the interpretation of survival and death patterns.^39^, ^40^, ^41^ Finally, our results have been not externally validated. A lack of external validation hinders clinical uptake.

## 5 CONCLUSION

In summary, the higher risk of cervical cancer death and metastasis in NECC patients compared with SCC and ADC persisted longer when it comes to follow‐up strategies. The visualized and practical nomogram we built succeeded in distinguishing high‐risk patients for death. Our findings can provide important guidelines for improving survival outcomes and life quality in NECC patients.

## AUTHOR CONTRIBUTIONS

**Haijuan Yu:** Data curation (equal); investigation (equal); methodology (equal); writing – original draft (equal). **Yongtian Lin:** Conceptualization (equal); data curation (equal). **Jie Lin:** Software (equal); writing – review and editing (equal). **Ning Xie:** Formal analysis (equal). **Linying Liu:** Formal analysis (equal). **Sufang Deng:** Formal analysis (equal). **Yang Sun:** Supervision (equal); writing – original draft (equal); writing – review and editing (equal).

## FUNDING INFORMATION

This work was supported by the Major Scientific Research Program for Young and Middle‐aged Health Professionals of Fujian Province, China (Grant No. 2022ZQNZD008).

## CONFLICT OF INTEREST STATEMENT

The authors declare that the research was conducted in the absence of any commercial or financial relationships that could be construed as a potential conflict of interest.

## ETHICS STATEMENT

There is no need for the ethics committee's approval due to the public availability of the SEER database.

## Supporting information


Data S1.

## Data Availability

The datasets presented in this study can be found in online repositories. The names of the repository/repositories and accession number(s) can be found in the article/supplementary material.
