# Supplementary material for: Causes of death analysis and the prognostic model construction in neuroendocrine carcinoma of the cervix: A SEER‐based study
Source: Cancer Med. 2024 Aug 9;13(15):e70066. doi: 10.1002/cam4.70066 (PMC11310552; doi:10.1002/cam4.70066)
Supplement: Supplementary file 1 — Data S1. [file CAM4-13-e70066-s001.docx]

**Supplementary**[**Materials**](https://www.ncbi.nlm.nih.gov/pmc/articles/PMC10807636/#cam46794-supitem-0007)

**Statistical analysis of** [**nomogram development**](https://pubmed.ncbi.nlm.nih.gov/37806946/)

Univariate and multivariate Cox regression analyses were performed, and variables with *P* < 0.05 in both univariate and multivariate Cox regression were identified as independent risk factors. The 1-/3‐/5‐year overall survival (OS) probabilities were estimated using the nomogram developed according to the independent predictors. We used VIF (variance inflation factor) to assess multicollinearity among variables. A VIF >10 was considered to indicate highly correlated variables, which were thus removed from the input data set. Finally, a concordance index (C-index), and a receiver operating characteristic (ROC) curve calculated by bootstrapping were constructed to assess the predictive performance of the nomogram. A Calibration curve was used to evaluate calibrating ability and a decision curve analysis (DCA) was used to assess the clinical benefits. DCA is a method for evaluating the clinical benefit of alternative models and was applied to nomograms by quantifying net benefits at different threshold probabilities.

[**Supplementary Table S1**](https://www.ncbi.nlm.nih.gov/pmc/articles/PMC10267371/#SM3) VIF of all variables of patients with NECC

| Variable | VIF |
| --- | --- |
| **Age (years)** |  |
| <40 | Reference |
| ≥40 | 1.1802 |
| **Race** |  |
| White | Reference |
| Black | 1.5521 |
| Others | 1.5052 |
| Unknown | 1 |
| **FIGO stage** |  |
| I | Reference |
| II | 22.445 |
| III | 54.004 |
| IV | 45.067 |
| **Tumor size** |  |
| ≤4cm | Reference |
| ＞4cm | 1.2284 |
| Unknown | 1.528 |
| **Regional lymph node involvement** |  |
| Yes | Reference |
| No | 1.7799 |
| Unknown | 2.1263 |
| **Distant metastasis** |  |
| Yes | Reference |
| No | 1.4178 |
| **Surgery** |  |
| Yes | Reference |
| No/Unknown | 1.554 |

[**Supplementary Table S2**](https://www.ncbi.nlm.nih.gov/pmc/articles/PMC10267371/#SM3) The VIF of the remaining variables in NECC patients, following distant metastasis was eliminated.

| Variable | VIF |
| --- | --- |
| **Age (years)** |  |
| <40 | Reference |
| ≥40 | 1.1543 |
| **Race** |  |
| White | Reference |
| Black | 1.5501 |
| Others | 1.502 |
| Unknown | 1 |
| **FIGO stage** |  |
| I | Reference |
| II | 1.659 |
| III | 1.2201 |
| IV | 1.8857 |
| **Tumor size** |  |
| ≤4cm | Reference |
| ＞4cm | 1.2304 |
| Unknown | 1.485 |
| **Regional lymph node involvement** |  |
| Yes | Reference |
| No | 1.7836 |
| Unknown | 2.1266 |
| **Surgery** |  |
| Yes | Reference |
| No/Unknown | 1.4132 |

[**Supplementary Table S3**](https://www.ncbi.nlm.nih.gov/pmc/articles/PMC10267371/#SM3) The VIF of the remaining variables in NECC patients, following FIGO stage was eliminated.

| Variable | VIF |
| --- | --- |
| **Age (years)** |  |
| <40 | Reference |
| ≥40 | 1.1476 |
| **Race** |  |
| White | Reference |
| Black | 1.5226 |
| Others | 1.4993 |
| Unknown | 1 |
| **Tumor size** |  |
| ≤4cm | Reference |
| ＞4cm | 1.2232 |
| Unknown | 1.3936 |
| **Regional lymph node involvement** |  |
| Yes | Reference |
| No | 1.7325 |
| Unknown | 1.5668 |
| **Distant metastasis** |  |
| Yes | Reference |
| No | 1.2263 |
| **Surgery** |  |
| Yes | Reference |
| No/Unknown | 1.3375 |

[**Supplementary Table S4**](https://www.ncbi.nlm.nih.gov/pmc/articles/PMC10267371/#SM3) Univariate and multivariate analysis of overall survival of NECC with the remaining variables following FIGO stage was eliminated.

|  | Univariate analysis | |  | Multivariate analysis | |
| --- | --- | --- | --- | --- | --- |
|  | Hazard ratio (95% CI) | P value |  | Hazard ratio (95% CI) | P value |
| Age (years) |  |  |  |  |  |
| <40 | Reference |  |  | Reference |  |
| ≥40 | 1.652 (1.277 - 2.138) | < 0.001 |  | 1.284 (0.973 - 1.695) | 0.077 |
| Race |  |  |  |  |  |
| White | Reference |  |  |  |  |
| Black | 1.716 (1.165 - 2.527) | 0.006 |  | 1.502 (1.018 - 2.216) | 0.041 |
| Others | 0.973 (0.707 - 1.339) | 0.866 |  | 1.031 (0.743 - 1.429) | 0.856 |
| Unknown | 0.000 (0.000 - Inf) | 0.939 |  | 0.000 (0.000 - Inf) | 0.946 |
| Pathological type |  |  |  |  |  |
| SCNEC | Reference |  |  |  |  |
| Non-SCNEC | 0.980 (0.604 - 1.592) | 0.936 |  |  |  |
| Unknown | 1.009(0.766 - 1.329) | 0.948 |  |  |  |
| Median household income |  |  |  |  |  |
| <$75,000 | Reference |  |  |  |  |
| ≥$75,000 | 1.114 (0.864 - 1.435) | 0.406 |  |  |  |
| Unknown | 1.317 (0.871 - 1.990) | 0.192 |  |  |  |
| Rural-Urban Continuum Code |  | 0.353 |  |  |  |
| Counties in metropolitan areas | Reference |  |  |  |  |
| Nonmetropolitan counties | 1.135 (0.799 - 1.612) | 0.479 |  |  |  |
| Unknown | 1.232 (0.843 - 1.800) | 0.280 |  |  |  |
| FIGO 2018 |  |  |  |  |  |
| I | Reference |  |  | - |  |
| II | 1.806 (1.113 - 2.930) | 0.017 |  | - | - |
| III | 2.351 (1.651 - 3.349) | < 0.001 |  | - | - |
| IV | 4.427 (3.200- 6.125) | < 0.001 |  | - | - |
| Tumor size |  |  |  |  |  |
| ≤4cm | Reference |  |  | Reference |  |
| ＞4cm | 1.795 (1.250 - 2.577) | 0.006 |  | 1.323 (0.723 - 1.604) | 0.082 |
| Unknown | 2.328 (1.660 - 3.263) | < 0.001 |  | 1.319 (0.901 - 1.933) | 0.450 |
| Regional lymph node involvement |  |  |  |  |  |
| Yes | Reference |  |  | Reference |  |
| NO | 0.518 (0.387 - 0.693) | < 0.001 |  | 1.013 (0.965 - 1.813) | 0.944 |
| Unknown | 1.127 (0.840 - 1.512) | 0.426 |  | 0.878 (0.625 - 1.232) | 0.789 |
| Distant metastasis |  |  |  |  |  |
| Yes | Reference |  |  | Reference |  |
| NO | 0.335 (0.261 - 0.430) | < 0.001 |  | - | - |
| Surgery |  |  |  |  |  |
| Yes | Reference |  |  | Reference |  |
| No/Unknown | 2.256 (1.808 - 2.815) | < 0.001 |  | 1.722 (1.289 - 2.301) | <0.001 |
| Radiotherapy |  |  |  |  |  |
| Yes | Reference |  |  |  |  |
| No/Unknown | 1.025 (0.803- 1.308) | 0.842 |  |  |  |
| Chemotherapy |  |  |  |  |  |
| Yes | Reference |  |  |  |  |
| No/Unknown | 1.181 (0.903 - 1.545) | 0.224 |  |  |  |


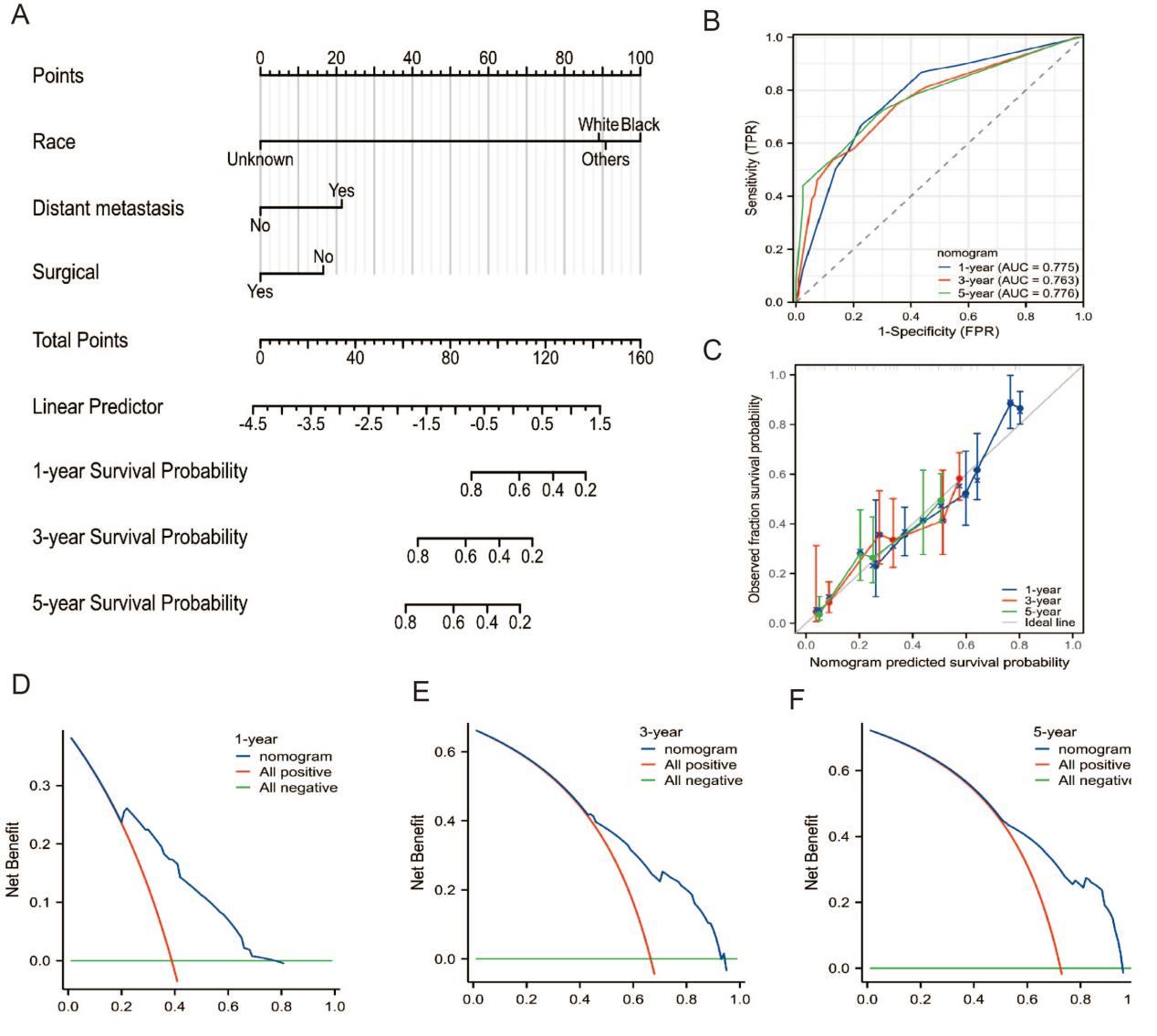


**Supplementary** **Figure S1.** The construction and validation of Nomograms with the remaining variables following FIGO stage was eliminated. (A) Nomogram model predicting the 1-, 3- and 5-year OS in NECC patients. The nomogram is used by summing all points identified on the scale for each variable. The total points projected on the bottom scales indicate the probabilities of 1-, 3- and 5-year survival. (B) ROC curves for predicting 1-, 3- and 5-year OS. (C) The calibration curves for predicting 1-, 3- and 5-year OS in NECC patients. (D-F) Decision curve analysis (DCA) for the nomograms in prediction of 1-, 3- and 5-year OS in NECC patients.
